# The olfactory Olfr-78/51E2 receptor interacts with the adenosine A_2A_ receptor. Effect of menthol and 1,8-cineole on A_2A_ receptor-mediated signaling

**DOI:** 10.3389/fphar.2023.1108617

**Published:** 2023-05-17

**Authors:** Jaume Lillo, Irene García-Pérez, Alejandro Lillo, Joan Serrano-Marín, Eva Martínez-Pinilla, Gemma Navarro, Rafael Franco

**Affiliations:** ^1^ Department of Biochemistry and Physiology, School of Pharmacy and Food Science, Universitat de Barcelona, Barcelona, Spain; ^2^ CiberNed, Network Center for Neurodegenerative Diseases, National Spanish Health Institute Carlos III, Madrid, Spain; ^3^ Molecular Neurobiology Laboratory, Department of Biochemistry and Molecular Biomedicine, Faculty of Biology, Universitat de Barcelona, Barcelona, Spain; ^4^ Department of Morphology and Cell Biology, Faculty of Medicine, University of Oviedo, Asturias, Spain; ^5^ Instituto de Neurociencias del Principado de Asturias (INEUROPA), Asturias, Spain; ^6^ Instituto de Investigación Sanitaria del Principado de Asturias (ISPA), Asturias, Spain; ^7^ Institut de Neurociències, Universitat de Barcelona, Barcelona, Spain; ^8^ School of Chemistry, Universitat de Barcelona, Barcelona, Spain

**Keywords:** G protein-coupled receptor, heteromer, receptor-receptor interactions, signaling, adenosine receptor, olfactory receptor, neuroprotection

## Abstract

Heteromer formation is unknown for the olfactory family of G protein-coupled receptors (GPCRs). We here identified, in a heterologous system, heteromers formed by the adenosine A_2A_ receptor (A_2A_R), which is a target for neuroprotection, and an olfactory receptor. A_2A_R interacts with the receptor family 51, subfamily E, member 2 (OR51E2), the human ortholog of the mouse Olfr-78, whose mRNA is differentially expressed in activated microglia treated with adenosine receptor ligands. Bioluminescence resonance energy transfer (BRET) assays were performed in HEK-293T cells expressing the human version of the receptors, OR51E2 and A_2A_R, fused, respectively, to *Renilla luciferase* (RLuc) and the yellow fluorescent protein (YFP). BRET data was consistent with a receptor-receptor interaction whose consequences at the functional level were measured by cAMP level determination in CHO cells. Results showed an olfactory receptor-mediated partial blockade of G_s_ coupling to the A_2A_R, i.e., the effect of the A_2A_R selective agonist on intracellular levels of cAMP was significantly reduced. Two odorants, menthol and 1,8-cineole, which failed to show G_olf_-mediated OR51E2 activation because they did not increase cytosolic cAMP levels, reduced the BRET readings in cells expressing A_2A_R-YFP and OR51E2-Rluc, most likely suggesting a conformational change of at least one receptor. These odorants led to an almost complete block of A_2A_R coupling to G_s_.

## Introduction

The olfactory receptor (OLFR) gene family is the largest in the mammalian genome (Buck and Axel, 1991), suggesting relevance of olfaction for survival and Evolution [see (Snyder et al., 1988; Dryer and Berghard, 1999; Gaillard et al., 2004) for review]. Despite the expression of OLFRs in different systems, their function outside the olfactory system is unknown. OLFR nomenclature for a given organism, for instance mouse, is different from that for others (see https://www.orthodb.org/?ncbi=1&query=259049). The proteins encoded by the *OLFR* genes belong to the well-studied superfamily of G protein-coupled receptors (GPCRs). However, there are two main reasons that explain the paucity of data relative to OLFRs in contrast to the large amount of information for the remaining members of class A GPCRs: i) the lack of pharmacological tools (agonists/antagonists), and ii) the difficult expression in heterologous systems (Alexander et al., 2021). The lack of tools has delayed the demonstration of whether OLFRs may be involved in receptor-receptor interactions as has been confirmed for other GPCRs, namely, for class C, class A, and taste receptors (Gines et al., 2000; Duthey et al., 2002; Hillion et al., 2002; Kniazeff et al., 2011; Kuhn and Meyerhof, 2013; Brugarolas et al., 2014; Fujita et al., 2014; Franco et al., 2016; Pin and Bettler, 2016).

Adenosine A_2A_ receptor (A_2A_R), a class A GPCR, is considered a target for neuroprotection. In the central nervous system, the receptor is widely expressed in striatal neurons, although it is also present in neurons in other brain regions as well as glia. In the microglia surrounding brain lesions of Alzheimer’s disease patients, the receptor is upregulated (Angulo et al., 2003). Relevant to therapeutic drug development has been the approval of istradefylline, a selective A_2A_R antagonist, as a first-in-class drug for the treatment of Parkinson’s disease (Jenner et al., 2009; Jenner et al., 2021; Mizuno et al., 2013; Suzuki et al., 2017; Berger et al., 2020; Mori et al., 2022). Istradefylline targets the A_2A_R which often interacts with other GPCRs on the cell surface as dopamine and cannabinoid receptors (see https://www.gpcr-hetnet.com/ for more interactions involving the receptors; accessed on 13 November 2022). The formation of such complexes is physiologically relevant since heteromers display functional properties that are not possible when receptors act as monomers/homomers (Ciruela et al., 2006; Fuxe et al., 2007; Brugarolas et al., 2014; Franco et al., 2018; Franco et al., 2021; Gallo et al., 2019).

On the homo/heteromerization potential of OLFRs, this sentence in a 2005 paper still holds true: “*Given the large number of OLFRs in mammals, the specificity and functional importance if heteromerization of the vast majority of receptors in this family remain to be determined*” (Prinster et al., 2005). The present article, whose objective was to search for interactions involving OLFRs, has taken advantage of the results of a transcriptomic study (data in preparation) showing that an OLFR, coded by the *Olfr-78* gene (mouse nomenclature), is differentially expressed when mouse primary activated microglial cells are treated with A_2A_R antagonists. Therefore, we reasoned that a first approach to the possibility that OLFRs could form receptor-receptor complexes was to determine whether this specific OLFR was capable of interacting with the A_2A_R in a heterologous system. Although the expression and relevance of OLFRs in general and of Olfr-78 in particular outside the olfactory system has not been widely studied, it was reported three years ago that the Olfr-78 gene product is expressed in the intestine and is involved in the response to colitis (Kotlo et al., 2020). Here, we have used bioluminescence energy transfer (BRET) assays to discover, in a heterologous expression system, a direct interaction between the human version of the A_2A_R and the *Homo sapiens* ortholog of Olfr-78, namely, the olfactory 51E2 receptor (OR51E2). In both cells expressing the A_2A_R and cells expressing the A_2A_-51E2 receptor heteromer, the effect of two odorants whose specific receptors have yet to be identified, menthol and 1,8-cineole, was studied by measuring intracellular cAMP levels.

## Methodology

### Reagents

CGS21680 (CAS: 124431-80-7) was purchased from Tocris Bioscience (Bristol, United Kingdom). Menthol (CAS: 89-78-1) and 1,8-cineole (CAS: 470-82-6) were purchased from Sigma-Aldrich (St. Louis, MO, United States).

### Expression vectors

The cDNA for the human A_2A_ receptor (hA_2A_R) was cloned in pcDNA3.1 and amplified without their stop codons using sense and antisense primers. The primers, harbored either unique EcoRI and BamHI sites to clone hA_2A_R, were subcloned to a pEYFP-containing vector to be in frame with the sequence of a yellow fluorescent protein (pEYFP-N1; Clontech, Heidelberg, Germany) and the green fluorescent protein 2 (pGFP^2^-N3; Clontech, Heidelberg, Germany). The plasmid coding for the fusion protein hOR51E2-RLuc (hOR51E2-RLuc) was provided by VectorBuilder (Chicago, IL, United States); the sequence for OR51E2 used to prepare the plasmid pRP[Exp]-CAG > hOR51E2[NM_030774.3](ns):3xGGGGS:Rluc (Vector IDVB220426-1096mmj) corresponds to that of [NM_030774.3] and may be found in https://www.ncbi.nlm.nih.gov/gene/?term=81285 (accessed on 22 November 2022).

### Cell culture and transient transfection

Human embryonic Kidney HEK-293T (batch 612968), acquired from the American-Type Culture Collection (ATCC, Manassas, VA, United States), and Chinese hamster ovary (CHO) cells were amplified and the aliquots stored in liquid nitrogen. Cells from each aliquot were used until passage 19. Cells were grown in Dulbecco’s modified Eagle’s medium (DMEM) (Gibco, Waltham, MA, United States) supplemented with 2 mM L-glutamine, 100 U/mL penicillin/streptomycin, MEM non-essential amino acid solution (1/100) and 5% (v/v) heat-inactivated fetal bovine serum (FBS) (all supplements were from Invitrogen, Paisley, Scotland, United Kingdom), and maintained in a humid atmosphere of 5% CO_2_ at 37°C. Cells were transiently transfected with the corresponding cDNAs using the PEI (PolyEthylenImine, Sigma-Aldrich, St. Louis, MO, United States) method as previously described (Carriba et al., 2008; Navarro et al., 2012). Four h after transfection, growth medium was replaced by complete medium. Experiments were carried out 48 h later.

### Bioluminescence resonance energy transfer assays

HEK-293T cells growing in 6-well plates were transiently cotransfected with a constant amount of cDNA encoding for hOR51E2-RLuc (1.5 μg) and with increasing amounts of cDNA for hA_2A_R-YFP (0.2–4 μg). 48 h post-transfection, cells were washed twice in quick succession with HBSS (137 mM NaCl; 5 mM KCl; 0.34 mM Na_2_HPO_4_; 0.44 mM KH_2_PO_4_; 1.26 mM CaCl_2_; 0.4 mM MgSO_4_; 0.5 mM MgCl_2_ and 10 mM HEPES, pH 7.4) supplemented with 0.1% glucose (w/v), detached by gently pipetting and resuspended in the same buffer. Cells were counted using an automatic counting device (Benchmark QuadCount Automated Cell Counter, Sigma-Aldrich) and used when viability, as assessed by 4′,6-diamidino-2-phenylindole (DAPI) staining, was >95%. To quantify YFP-fluorescence expression, cells were distributed (20 μg protein) in 96-well plates (black plates with a transparent bottom; Porvair, Leatherhead, United Kingdom). Fluorescence was read using a FluoStar Optima fluorimeter (BMG Labtechnologies, Offenburg, Germany) equipped with a high-energy xenon flash lamp, reading at 530 nm. YFP-fluorescence expression was determined as the fluorescence of the sample minus the fluorescence of cells only expressing protein-RLuc. Odorant action was measured using sealed 96-well plates having odorants, menthol or 1,8-cineole [different white plates (Porvair) for each], at a concentration of 5 M in the peripheral wells (100 µL/well) and leaving one file and one row without any cell/reagent. Cells and other reagents were placed in the rest of the wells. For BRET measurements, coelenterazine H (Molecular Probes, Eugene, OR) was added (5 µM final concentration) 15 min after sealing the plates with odorants, and 60 s later, readings were collected using a Mithras LB 940 (Berthold, Bad Wildbad, Germany), which allowed the integration of the signals detected in the short-wavelength filter at 485 nm (475–495 nm) and the long-wavelength filter at 530 nm (520–540 nm). The net BRET is defined as [(long-wavelength emission)/(short-wavelength emission)]-Cf where Cf corresponds to [(long-wavelength emission)/(short-wavelength emission)] for the RLuc construct expressed alone in the same experiment. To quantify receptor-RLuc expression, luminescence readings were collected 10 min after 5 μM coelenterazine H addition. An equivalent plate was prepared, sealed and processed, but in the absence of odorants. The BRET curves were fitted by non-linear regression using the GraphPad Prism software (San Diego, CA, United States). BRET values are given as milli BRET units (mBU: 1000 × net BRET).

### cAMP level determination

CHO cells transfected or not with plasmids coding for receptor/fusion proteins were seeded in 6-well plates. Two hours before initiating the experiment, cell medium was substituted by non-supplemented DMEM medium. Then, cells were detached, re-suspended in non-supplemented medium containing 50 μM zardaverine, and plated in 384-well microplates (2,500 cells/well). Plates were described as in the previous section. Cells were exposed to odorants for 15 min prior A_2A_R activation using 100 nM of a selective agonist, CGS21680; 15 min later the process was stopped by the addition of the Eu-cAMP tracer and the ULight-cAMP monoclonal antibody prepared in the “cAMP detection buffer” provided by PerkinElmer. All steps were performed at 25°C. Homogeneous time-resolved fluorescence energy transfer (HTRF) measures were performed 60 min after incubation using the LanceUltra cAMP kit (PerkinElmer, Waltham, MA, United States). Fluorescence at 665 nm was analyzed on a PHERAstar Flagship microplate reader equipped with an HTRF optical module (BMGLab technologies, Offenburg, Germany). The effect of CGS21680 was calculated respect to the basal levels in each condition: vehicle, exposed to menthol or exposed to 1,8-cineole.

## Results

### The OR51E2 and the A_2A_R may establish direct interactions in living cells

**FIGURE 1:**
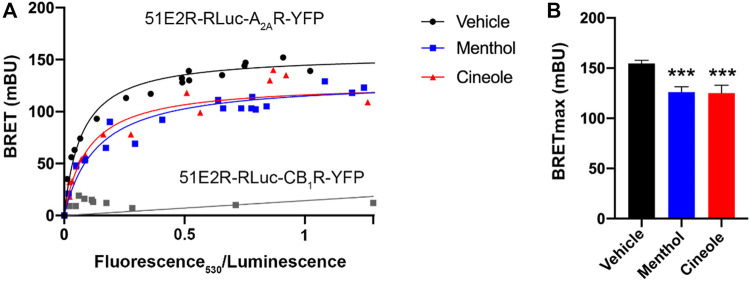
Bioluminescence resonance energy transfer (BRET) assays. **(A)** BRET assays were performed in HEK-293T cells cotransfected with a constant amount of cDNA for hOR51E2-RLuc (1.5 μg) and increasing amounts of cDNA for hA_2A_R-YFP (0.2–4 μg). Similar assays were performed in cells placed in an atmosphere containing menthol or 1,8-cineole (See *Methodology* for experimental details). HEK-293T cells transfected with a constant amount of hOR51E2R-Rluc cDNA (1.5 μg) and increasing amounts of cannabinoid CB_1_ receptor-YFP cDNA (0.2–4 μg) were used to provide a negative control. Net BRET (in milli BRET units) is plotted against the ratio: net YFP fluorescence/luminescence; net YFP fluorescence (530 nm) is obtained subtracting from the total value of 530 nm readings the autofluorescence of cells expressing the donor protein but not the YFP-containing acceptor. **(B)** Effect of odorants on BRET_max_ in cells expressing hOR51E2-RLuc and hA_2A_R-YFP. BRET data are expressed as the mean ± S.E.M of 8 different experiments performed in duplicates. mBU: milliBRET units. Significant differences were analyzed by a one-way ANOVA followed by *post-hoc* Bonferroni’s test. ***p < 0.001 versus BRET_max_ in untreated (vehicle) cells.

HEK-293T cells were transiently transfected with cDNAs coding for the human version of the receptors fused to either a BRET donor (RLuc) or acceptor (YFP). The BRET signal was saturable in cells expressing a constant amount of hOR51E2-RLuc and increasing amounts of hA_2A_R-YFP (Figure 1A), thus indicating a direct interaction between the two proteins. The BRET parameters were: BRET_max_ = 155 ± 3 mBU and BRET_50_ = 0.072 ± 0.007. In contrast, no interaction was detected in cells expressing hOR51E2-RLuc and the cannabinoid CB_1_ receptor-YFP fusion protein because the BRET relationship was linear (Figure 1A). Two odorants, menthol and 1,8-cineole, whose specific OLFRs are unknown, led to a reduction of the BRET signal in cells expressing hOR51E2-RLuc and hA_2A_R-YFP. As shown in Figure 1B, BRET_max_ in the presence of odorants was significantly reduced. These results suggest conformational changes in the h51E2/A_2A_ receptor heteromer leading to a variation in the distance between donor and acceptor, i.e., menthol or 1,8-cineole increased the distance between RLuc and YFP fused to, respectively, the OR51E2 and the A_2A_R. The possibility of a slight reduction in the number of heteromers caused by odorants cannot be ruled out.

### A_2A_R-mediated signaling in CHO cells expressing the A_2A_R and in cells also expressing the 51E2/A_2A_ receptor heteromer

**FIGURE 2:**
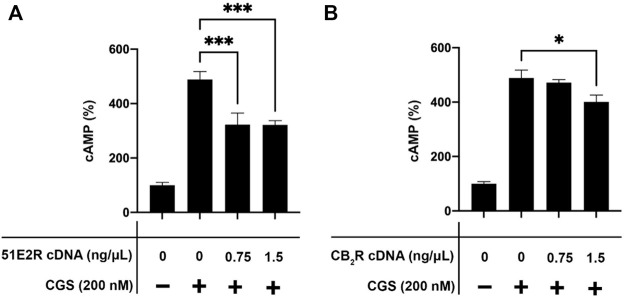
Effect of CGS21680 in cells expressing the A_2A_R-GFP^2^ and either the cannabinoid CB_2_R or the OR51E2. In all cases cells were transiently expressing the A_2A_R-GFP^2^. When indicated, cells were cotransfected using two different amounts of cDNA coding for the CB_2_R or for OR51E2. cAMP levels were determined, as described in Methodology, in cells treated with 200 nM CGS21680 (CGS), the selective A_2A_R agonist. **(A)** Effect of CGS21680 in cells transfected with a constant amount of the hA_2A_R-GFP^2^ cDNA (1.5 ng/μL) and increasing amounts of the OR51E2 cDNA (0–1.5 ng/μL). **(B)** Effect of CGS21680 in cells transfected with a constant amount of the hA_2A_R-GFP^2^ cDNA (1.5 ng/μL) and increasing amounts of the CB_2_R cDNA (0–1.5 ng/μL). Values are the mean ± S.E.M of 4 independent experiments in triplicates. One-way ANOVA followed by Bonferroni’s multiple comparison *post-hoc* test were used for statistical analysis. *p < 0.01; ***p < 0.001 compared with the condition expressing hA_2A_R-GFP^2^ treated with CGS21680.

In preliminary assays, we discovered that the effect of A_2A_R agonists was reduced when the OR51E2 was coexpressed. To have a readout of the expression of the hA_2A_R we decided to use the hA_2A_R-GFP^2^ fusion protein for the signaling assays. The adenosine receptor couples to heterotrimeric G_s_ proteins and, therefore, its activation by agonists leads to activation of adenylate cyclase and, subsequently, to increases in intracellular cAMP levels. The increase of intracellular cAMP concentration in response to 200 nM CGS21680, a selective A_2A_R agonist, was significant in CHO cells expressing the hA_2A_R-GFP^2^ (Figure 2). The effect was reduced when the OR51E2 was also expressed thus suggesting a blockade of A_2A_R/G_s_-mediated signaling upon formation of the adenosine-olfactory receptor complex. The decrease in the response was similar when cells were transfected with 0.75 ng/μL and with 1.5 ng/μL cDNA for the OR51E2 (Figure 2A). Assessment of the expression of the hA_2A_R-GFP^2^ by means of fluorescence readings showed that the expression of the protein was similar in all cases (between 5800 and 6400 relative units) with the highest value in cells transfected with the cDNA for the hA_2A_R-GFP^2^ and 1.5 ng/μL cDNA for the OR51E2. To further confirm that the partial blockade was specifically due to the coexpression of the OLFR, similar assays were performed in cells expressing the hA_2A_R-GFP^2^ and the cannabinoid CB_2_ receptor (CB_2_R), which forms heteromers with each other (Franco et al., 2019; Franco et al., 2019). Coexpression of adenosine and cannabinoid receptors using 0.75 ng/μL cDNA for the CB_2_R did not lead to any significant decrease in the response to CGS21680; increasing the expression level of the CB_2_R by using 1.5 ng/μL cDNA led to a decrease in signaling that was significant but much less than that achieved by OR51E2 expression. These results suggest that expression of the OR51E2 and the formation of heteromers with the A_2A_R leads to a significant reduction in the response triggered by the A_2A_R agonist.

**FIGURE 3:**
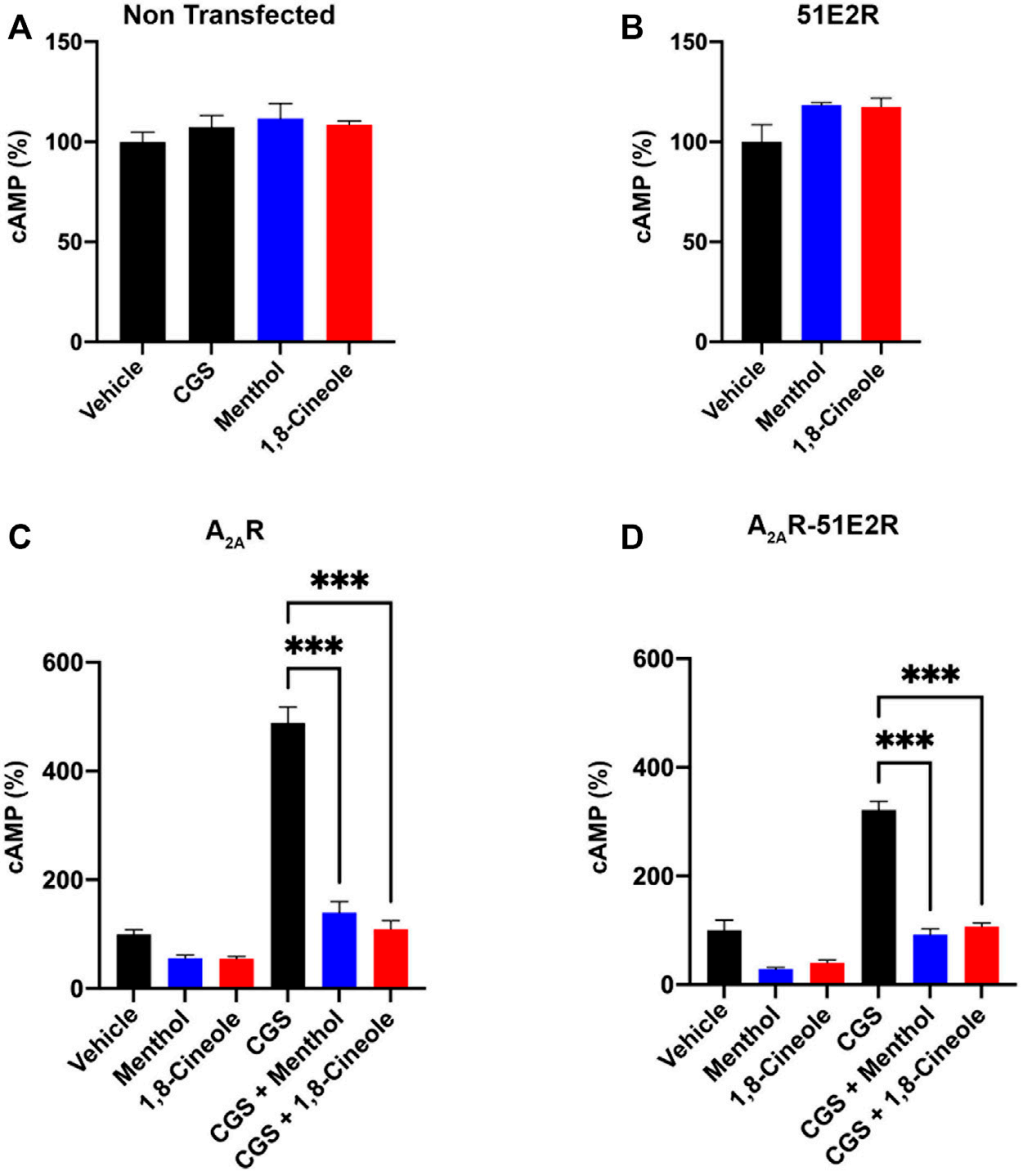
Effect of menthol or 1,8-cineole in A_2A_R-mediated signaling. cAMP levels were determined, as described in Methodology, in CHO cells treated with 200 nM CGS21680 (CGS), the selective A_2A_R agonist. **(A)** Lack of effect of CGS21680, menthol or 1,8-cineole upon cAMP levels in non-transfected cells. **(B)** Lack of effect of menthol or 1,8-cineole in cells expressing the OR51E2 (100% indicates the [cAMP] obtained in the presence of vehicle). **(C)** Effect of CGS21680 in the presence or absence of menthol or 1,8-cineole in cells expressing the A_2A_R (100% indicates the [cAMP] obtained in the presence of vehicle). **(D)** Effect of CGS21680 in the presence or absence of menthol or 1,8-cineole in cells coexpressing A_2A_ and 51E2 receptors (100% indicates the [cAMP] obtained in the presence of vehicle). Values are the mean ± S.E.M of 4 independent experiments in triplicates. One-way ANOVA followed by Bonferroni’s multiple comparison *post-hoc* test were used for statistical analysis. ***p < 0.001 compared with treatment with CGS21680.

The effect of the odorants was tested in cells expressing the hA_2A_R, the OR51E2 or both. First, it was assayed whether the presence of menthol or 1,8-cineole affected the intracellular cAMP levels of non-transfected cells. The results showed that this is not the case since CGS21680 did not elicit any response in these cells (Figure 3A). Furthermore, we found that none of the odorants were able to participate in G_olf/s_-mediated signaling in CHO cells expressing the OR51E2. (Figure 3B). Finally, menthol and 1,8-cineole decreased the basal levels of intracellular cAMP in cells expressing the hA_2A_R, alone or in combination with the OR51E2 (Figures 3C, D). Interestingly, the effect of CGS21680 was markedly reduced, almost neglected, in the cells previously exposed to menthol or 1,8-cineole. This result was similar in cells expressing the A_2A_R or coexpressing the two receptors (Figures 3C, D).

## Discussion

Despite constituting a highly populated family of the mammalian proteome, OLFRs are poorly characterized due to the lack of pharmacological tools. The sequences of the genes and the primary structure of OLFRs show that these proteins belong to the GPCR superfamily. Also of note, the heterotrimeric G proteins that mediate olfaction are known as “G_olf_” (“olf” for olfactory). G_olf_ proteins were first discovered in sensory neurons of the olfactory epithelium (Jones and Reed, 1989); shortly thereafter they were identified in the basal ganglia where they were supposed to mediate dopaminergic neurotransmission (Drinnan et al., 1991). Interestingly, the A_2A_R is enriched in the basal ganglia where it colocalizes and activates G_olf_ (Kull et al., 2000). In fact, there are two G proteins to which the A_2A_R may couple: G_olf_ and G_s_ (Alexander et al., 2019). Given the findings of this study, it is tempting to speculate whether the coupling of A_2A_R to G_olf_ is direct or mediated by heteromerization with G_olf_-coupled OLFRs.

Unlike other GPCRs for which the physiological agonist is well known, there is little indication of physiological agonists and/or volatile compounds capable of activating OLFRs. One of the unknown aspects of these receptors is whether they are able to establishing receptor-receptor interactions, as it is the case with members of other GPCR families (Borroto-Escuela et al., 2014). In fact, the relevance of GPCR heteromerization to understand the actual function of OLFRs, under physiological conditions, and their actual potential as targets for therapy is becoming clearer. The results in this paper show that neither menthol nor 1,8-cineole can engage the putative G_olf/s_ that could be coupled to the OR51E2 (Figure 3). The minimal changes observed by the action of menthol or 1,8-cineole were observed both in cells expressing the A_2A_R and in cells expressing the A_2A_R and the OR51E2. Therefore, these two odorants are unlikely to directly interact with the OR51E2, unless the receptor is not coupled to G_olf_ but to another signal transduction pathway.

The main result of this paper is the demonstration that the human A_2A_R can interact with the human OR51E2 in a heterologous system. The discovery of this interaction is relevant as it shows, for the first time, that OLFRs can participate in receptor-receptor interactions as has already been shown for rhodopsin-like class A, for class C and for taste GPCRs (Borroto-Escuela et al., 2014). However, understanding the physiological significance of the finding will require further investigation. Our results prove that heteromer formation correlates with a sharp reduction of the G_s_-mediated signaling triggered by A_2A_R agonists. In some heteromeric settings, activation of one of the receptors is required for crosstalk between receptors and regulation of signal transduction. Within the 51E2/A_2A_ receptor heteromer, adenosinergic signaling is reduced even in the absence of OLFR activation. This result indicates that there are marked conformational changes in the structure of the A_2A_R when the OR51E2 is present. There are examples of regulation of signaling simply through the coexpression of two receptors. One is the heteromer formed by A_2A_R and another adenosine receptor, A_3_, which couples to G_i_. Within the A_2A_-A_3_ receptor heteromer, the A_3_R-mediated signaling is blocked when the two receptors are coexpressed; blockade is detectable both in a heterologous expression system and in primary striatal neurons from mouse brain (Lillo et al., 2020).

If the interaction has physiological relevance, there are two different scenarios that are not mutually exclusive. One involves the cells in which the A_2A_R might be expressed in the olfactory system. In the upper respiratory tract, both A_2A_ and A_2B_ adenosine receptors have been shown to regulate mucociliary clearance in the nose, where OLFRs are primarily expressed (Hua et al., 2013). The second scenario would consist of a non-olfactory system in which A_2A_R and OR51E2 are jointly expressed in a certain cell type. Hans Hatt is known for being the scientist who best understood the relevance of “ectopic” OLFRs, that is, receptors located outside the nose that exert non-olfactory actions. After intense work over decades, he and his colleagues have reviewed research suggesting roles of OLFRs outside the nose and speculating about their potential as therapeutic targets (Maßberg and Hatt, 2018; Lee et al., 2019). Using kidney cells, Hatt and others suggested that the mouse ortholog of OR51E2, Olfr78, might respond to propionate, which would then become an odorless physiological ligand for the receptor (Pluznick et al., 2013). Noteworthy, the Olfr-78 is highly expressed in the gastrointestinal system and its expression is reduced in two experimental models of colitis (Kotlo et al., 2020). In the same study it was proven that OR51E2 is expressed in human colon (Kotlo et al., 2020). Recently, this OLFR has been found in a variety of mouse central nervous system cells, including microglia and vasopressin/oxytocin neurons (Nakashima et al., 2022).

To the best of our knowledge, the OLFRs involved in the sense of odor produced by menthol or 1,8-cineole have not been identified. The experiments reported here with odorants also provided limited but relevant information. First, menthol and 1,8-cineole reduced heteromer formation and/or affected the conformation of the receptor-receptor complex, as detected by a significant decrease in BRET_max_ (Figure 1). This effect can be direct, on one of the receptors. As discussed above, menthol or 1,8-cineole do not appear to interact with the OR51E2. Conversely, it cannot be completely ruled out that odorants interact with the A_2A_R, as both were able to markedly reduce the G_s_-mediated response elicited by the A_2A_R agonist. Supporting this view is the negative regulation occurring in both cells expressing the A_2A_R and in cells coexpressing A_2A_ and 51E2 receptors. In summary, menthol and 1,8-cineole do not seem to activate the OR51E2, but they block G_s_-mediated signaling suggesting that a direct interaction of these odorants with the adenosine receptor is possible. Otherwise, these odorants could interact with the membrane components surrounding the heteromer, thus affecting its overall structure.

## Data Availability

The original contributions presented in the study are included in the article/Supplementary Materials, further inquiries can be directed to the corresponding authors.
